# The Impact of Endometriosis on the Health of Women 2016

**DOI:** 10.1155/2016/1747280

**Published:** 2016-11-01

**Authors:** Liselotte Mettler, Dietmar Schmidt, Peter Maher

**Affiliations:** ^1^Department of Obstetrics and Gynecology, University Clinics of Schleswig-Holstein, Arnold Heller Strasse 3/24, 24105 Kiel, Germany; ^2^Institute of Pathology, Gereonstr. 14a, 41747 Viersen, Germany; ^3^Department of Endosurgery, Mercy Hospital for Women, Heidelberg, VIC 3084, Australia

Originally described already more than 300 years ago endometriosis is an estrogen-dependent, chronic, inflammatory disease prevalent worldwide in 10–30% of women of reproductive age and beyond. Characterized by the growth of endometrium-like tissue in aberrant locations outside of the uterus, it is responsible for symptoms including chronic pelvic pain, inflammation, dysmenorrhea, dyspareunia, and subfertility that degrade quality of life of women significantly. In Germany the direct and indirect economic cost of endometriosis amount annually to 1.5 billion dollars, which today equalizes euros and this is elevated to 20 billion dollars/euros in the United States [1].

The awareness of endometriosis and adenomyosis in patients and doctors has changed and is continuously updated. The aim of this special issue in 2016 is to focus on several features in endometriosis, their research in particular molecular aspects as well as their clinical applications for surgical excisional treatment and for deliveries, and certain aspects of infertility. We have selected six papers, of which 2/3 deal with basic research aspects, and the other third highlights the clinical management and treatment of this enigmatic but not malignant disease.

In the first review article I. Jeung et al. deal with the decreased cytotoxicity of peripheral and peritoneal Natural Killer cells (NK cells) in endometriosis patients and, in a clinical review on the laparoscopic treatment of deep infiltrating endometriosis (DIE), which still remains a difficult confrontation for every surgeon, O. Triolo et al. compare the full thickness excision to pure shaving of such a lesion. While it is today accepted to surgically treat deep infiltrating endometriosis to the bowel and bladder by laparoscopy, the controversy whether to use full thickness excision or shaving remains and depends on the situation, the surgeon's decision, and the patent's wish.

Two research articles address the epidemiology of endometriosis in France by J. Cottenet et al. and certain demographic clinical features of endometrial polyps treated by hysteroscopy by Y. Zhang et al.

Concerning clinical and research aspects the other 2 papers focus on delivery after surgery of deeply infiltrating endometriosis by S. H. Enzelsberger et al. of Peter Oppelt's group of Linz, Austria, and on the negative finding of serum levels of the soluble factors sCD40L and CXCL1 for the diagnosis of endometriosis by the Viennese group of K. Walch et al. of Austria.

To conclude, we know that an early recognition of endometriosis in young girls can save years of suffering. Considering the current special issue we think the selected articles offer an ideal opportunity to update our knowledge on some new achievements to diagnose and treat this disease and its following steps as possible infertility and problems even at deliveries.

*Liselotte Mettler* *Dietmar Schmidt* *Peter Maher*
